# A Novel Cross-Priming Amplification-Based Assay for Tuberculosis Diagnosis in Children Using Gastric Aspirate

**DOI:** 10.3389/fmicb.2022.819654

**Published:** 2022-03-24

**Authors:** Shuting Quan, Tingting Jiang, Weiwei Jiao, Yu Zhu, Qiong Liao, Yang Liu, Min Fang, Yan Shi, Li Duan, Xiaomei Shi, Yacui Wang, Xue Tian, Chaomin Wan, Lin Sun, Adong Shen

**Affiliations:** ^1^Beijing Key Laboratory of Pediatric Respiratory Infection Diseases, Key Laboratory of Major Diseases in Children, Ministry of Education, National Clinical Research Center for Respiratory Diseases, National Key Discipline of Pediatrics (Capital Medical University), Beijing Pediatric Research Institute, Beijing Children’s Hospital, Capital Medical University, National Center for Children’s Health, Beijing, China; ^2^Baoding Children's Hospital, Baoding, China; ^3^West China Second Hospital, Sichuan University, Chengdu, China; ^4^The No. 1 People's Hospital of Liangshan Yizu Autonomous Prefecture, Liangshan, China

**Keywords:** child, diagnosis, gastric aspirate, tuberculosis, Ustar EasyNAT MTC assay, Xpert MTB/RIF

## Abstract

Low detection rates of *Mycobacterium tuberculosis* (MTB) by culture and smear microscopy prevent early diagnosis of tuberculosis (TB) in children. Therefore, developing rapid and accurate diagnostic techniques are critical to achieving the global aim of minimizing childhood TB. The present study was performed to evaluate the diagnostic effectiveness of the novel cross-priming amplification-based EasyNAT MTB complex assay (EasyNAT) in childhood TB. Five hundred and six children with suspected TB were enrolled from January 2018 to October 2021. Gastric aspirate (GA) samples were tested by bacterial culture, acid-fast bacillus microscopy, EasyNAT, Xpert MTB/RIF (Xpert), or Xpert MTB/RIF Ultra (Xpert Ultra). Among 239 children simultaneously tested by EasyNAT and Xpert methods, both assays showed similar sensitivities in total active TB cases [22.6% (31/137) vs. 26.3% (36/137), *p* = 0.441] and in bacteriologically confirmed TB cases [both 60.0% (9/15)]. The two assays presented similar specificities of 98.0% (100/102) and 99.0% (101/102), respectively (*p* = 1.000). Among 267 children who were simultaneously tested with EasyNAT and Xpert Ultra, Xpert Ultra demonstrated higher sensitivity than EasyNAT in total active TB cases [50.9% (89/175) vs. 30.3% (53/175), *p* < 0.001]. EasyNAT and Xpert Ultra yielded similar specificities, at 97.8% (90/92) and 100.0% (92/92), respectively (*p* = 0.155). These findings indicated that Xpert Ultra was superior to EasyNAT despite its higher cost and EasyNAT was not inferior to Xpert in the diagnosis of childhood TB using GA samples. EasyNAT may therefore be a suitable alternative diagnostic method for childhood TB based on its cost-effectiveness, speed, and accuracy.

## Introduction

According to the latest global tuberculosis (TB) report, there were 9.9 million new TB cases in 2020, with children accounting for 11% of these and 16% of TB-associated deaths globally. The higher proportion of child deaths and lower disease detection rate indicates that children have a poorer chance of receiving effective disease prevention and control than adults. In 2020, the estimated number of new TB cases in China was 842,000, but children comprised only 1% (World-Health-Organization, 2021a). The diagnosis and treatment of childhood TB are hampered by the paucibacillary nature of samples and the difficulty in collecting high-quality specimens. Therefore, bacteriological confirmation by *Mycobacterium tuberculosis* (MTB) culture and smear microscopy detects low positivity rates in children, reducing the early diagnosis and treatment of childhood TB. Thus, developing rapid and accurate diagnostic techniques are critical to achieving the global aim of minimizing TB in children.

Xpert MTB/RIF (Xpert) and its next-generation product, Xpert MTB/RIF Ultra (Xpert Ultra), are both recommended by the WHO for use as initial diagnostic tests for TB in children with signs and symptoms of pulmonary TB (PTB; World-Health-Organization, 2021b). Xpert and Xpert Ultra are both automated, cartridge-based molecular tests that permit rapid detection of MTB complex (MTC) and identification of rifampicin resistance (Sun et al., 2019). However, Xpert and Xpert Ultra are expensive and require sophisticated instruments. Thus, these techniques are not suitable for resource-limited areas, many of which may have high incidence of TB.

Isothermal amplification of nucleic acids can rapidly and efficiently amplify nucleic acid sequences at a constant temperature (Zhao et al., 2015). To date, many isothermal amplification technologies, including helicase-dependent amplification (Barreda-Garcia et al., 2016), multiple cross displacement amplification (Jiao et al., 2019), loop-mediated isothermal amplification (Sharma et al., 2020; Dayal et al., 2021), and cross-priming amplification (CPA), have been used to detect MTB. Among these, CPA is a powerful, innovative amplification method and has been used in the detection of various pathogens, such as infectious spleen and kidney necrosis virus, Salmonella enterica serovar Indiana, and *Staphylococcus aureus* (Qiao et al., 2015; Liu et al., 2018; Wang et al., 2018). A novel kit, the EasyNAT MTC assay (EasyNAT, Ustar, Biotechnologies Co. Ltd., China) based on the CPA technique, has been developed to diagnose TB in adults using sputum and has demonstrated good accuracy (Zhang et al., 2021). In this kit, two cross primers targeting the insertion sequence IS*6110* were designed to specifically detect MTC and improve sensitivity. The assay is carried out in a sealed tube to prevent cross-contamination and the DNA extraction, DNA purification, amplification, and detection of the target gene are performed in three separate chambers within the cartridge (Zhang et al., 2021). As a quick point-of-care test, the results can be reported within 2 h, which can provide early diagnosis for TB.

The recently published WHO communication on the management of TB in children and adolescents highlighted that evidence-based evaluations of diagnostic assays are needed in children especially in those younger than 10 years old to overcome the current shortfall in case detection (World-Health-Organization, 2021c). It was also suggested that ideal treatment decision algorithms could be tailored to be highly specific to each country’s settings and resources, and should consider different settings with varying access to diagnostic tests (World-Health-Organization, 2011a). Considering the rapid turnaround and low cost when compared with Xpert, the EasyNAT assay may represent a valuable tool for diagnosis of TB in children. This study enrolled children with suspected PTB and aimed to evaluate the diagnostic value of EasyNAT in childhood TB using gastric aspirate (GA) and compare the accuracy of EasyNAT with the established Xpert and Xpert Ultra methods.

## Materials and Methods

### Study Population

From January 2018 to October 2021, children aged 18 years or younger were enrolled in the study if they had (1) symptoms suggestive of TB, including, but not limited to, long-lasting fever, night sweats, and weight loss, and (2) positive chest radiograph changes.

The enrolled children were categorized into three groups (Graham et al., 2012) based on the final diagnosis. (1) Bacteriologically confirmed TB: positive results of MTB culture and/or microscopy; (2) probable TB: at least 1 sign and symptom AND X-ray abnormalities suggestive of tuberculosis AND at least 1 of the following: exposure history of active TB, clinical presentation improvement after anti-TB treatment, positive results of tuberculin skin test, or interferon-γ release assay; and (3) non-TB patients with respiratory tract infections (RTIs): symptomatic but not fitting the above criteria and confirmed evidence of bacterial or viral infection (Supplementary Table S1). The respiratory samples were tested by molecular commercial kits and/or bacterial culture. Patients who had previously received anti-TB treatment or being treated for more than 1 week were not included in this study.

This study was approved by the Medical Ethics Committee of Beijing Children’s Hospital, Capital Medical University. Written informed consent was obtained from the guardians of the patients.

### Procedures

GA samples were collected early in the morning after an overnight fast of at least 4 h. Children without the contraindications (including severe high blood pressure, esophageal stricture, esophageal tumor, heart failure, and upper gastrointestinal bleeding) can be collected GA using nasogastric tube. The nasogastric tube entered the stomach through the nose, and 2–8 ml of GA was drawn with a syringe. Each specimen was transported to the lab within 6 h of collection and stored at −80°C after aliquoting. The samples were then subjected to mycobacteria growth indicator tube (MGIT) 960 culture, microscopy testing, EasyNAT, Xpert, and Xpert Ultra assays.

According to clinical practice standards, MTB culture and acid-fast bacilli microscopy were tested on three consecutive days, while the molecular tests were performed once using the samples collected on the first day. Therefore, the children with bacteriologically confirmed TB were defined as those who were tested positively by MTB culture or acid-fast bacilli microscopy using the first samples collected.

### Culture and Smear

Two milliliters of GA sample were decontaminated with 2 ml N-acetyl-L-cysteine 2% sodium hydroxide for 15–20 min after sufficient vortexing. The mixture was then neutralized with sterile saline phosphate buffer (PBS) to a final volume of 45 ml and centrifuged at 3,000 × *g* for 15 min at 4°C. The pellet was resuspended in 1 ml of PBS and inoculated into the MGIT 960 system (Becton, Dickinson and Company, United States) and Lowenstein-Jensen solid medium.

The pellet was smeared on a slide for Ziehl-Neelsen acid-fast staining and examined by microscopy directly.

### Xpert and Xpert Ultra Assays

Two milliliters of GA sample were added to sodium hydroxide (final concentration, 0.75%) and vortexed at 5-min intervals for 15 min, followed by centrifugation at 4,000 × *g* for 15 min. The pellet was resuspended in 2 ml of the sample processing reagent and the mixture was transferred into Xpert or Xpert Ultra cartridges then loaded into the GeneXpert instrument. Both assays presented results in the same semiquantitative categories of high, medium, low, and very low. Additionally, a semiquantitative category of “trace” was introduced in the Xpert Ultra assay, which was designed to identify samples with the lowest number of targets for MTB. Invalid results were repeated. When a valid result was produced, the semiquantitative scale from each test was recorded.

### EasyNAT Assay

Two milliliters of GA sample were homogenized and digested using 3–4 ml 4% sodium hydroxide solution for 15 min at room temperature until fully liquified. After centrifugation at 4,000 × *g* for 10 min and then 12,000 rpm for 3 min, the pellet was resuspended with DNA extraction liquid premixed with the internal control before being added to the sample chamber of the cartridge. This assay evaluated the specimens as invalid, negative, positive, and no result. Invalid results were repeated and the valid results (negative or positive) were recorded.

### Statistical Analysis

SPSS version 25.0 (IBM, Armonk, NY, United States) was used for statistical analysis. Sensitivity and specificity were compared using the Chi-square test, while concordance between the different diagnostic tests was assessed using the Kappa test. Correlation of the semiquantitative scale of the tests was presented using Spearman’s rank correlation coefficients. *p* < 0.05 was considered as statistically significant.

## Results

### Patient Characteristics

**Figure 1:**
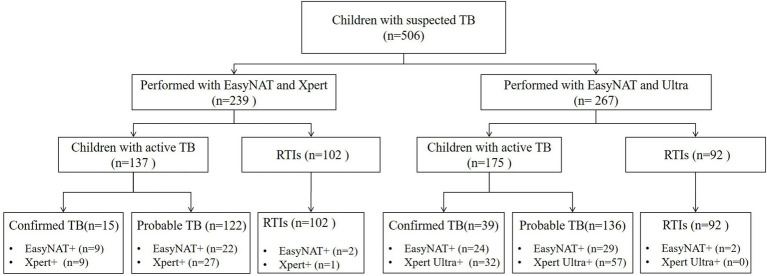
Flow chart of the study population. TB, tuberculosis; RTIs, respiratory tract infections; EasyNAT, EasyNAT MTC assay; Xpert, Xpert MTB/RIF; and Ultra, Xpert MTB/RIF Ultra.

In total, 506 children with suspected PTB were recruited. The median (interquartile range) age was 6.9 (2.4–11.1) years, with 199 (39.3%) cases under 5 years of age, and 328 (64.8%) cases under 10 years of age. All 506 enrolled children were classified into two groups: 239 children (including 137 children with TB and 102 children with RTIs) tested simultaneously with both EasyNAT and Xpert methods were enrolled in group 1 and 267 children (including 175 children with TB and 92 children RTIs) tested simultaneously by both EasyNAT and Xpert Ultra methods were enrolled in group 2. Figure 1 and Table 1 show the patient selection and their clinical characteristics.

**Table 1:** Main clinical characteristics of the study population.

Characteristic	Total (*n* = 506), *n* (%)	Part 1 (*n* = 239)			Part 2 (*n* = 267)		
		Bacteriologically confirmed TB (*n* = 15), *n* (%)	Probable TB (*n* = 122), *n* (%)	RTIs (*n* = 102), *n* (%)	Bacteriologically confirmed TB (*n* = 39), *n* (%)	Probable TB (*n* = 136), *n* (%)	RTIs (*n* = 92), *n* (%)
Age Mean (interquartile Range)	6.9 (2.4–11.1)	2.8 (1.4–12.4)	10.0 (7.0–12.8)	4.9(0.9–8.0)	7.0 (4.4–11.7)	8.5 (4.3–11.6)	4.8 (0.92–8.0)
full range	0.0–16.0	0.2–14.0	0.2–15.0	0.0–16.0	0.3–14.0	0.2–15.3	0.0–13.2
Gender							
Male	288(56.9)	8(53.3)	73(59.8)	62(60.8)	18(46.2)	73(53.7)	54(58.7)
Female	218(43.1)	7(46.7)	49(40.2)	40(39.2)	21(53.8)	63(46.3)	38(41.3)
Tuberculin skin test							
Positive	279(55.1)	12(80.0)	107(87.7)	17(16.7)	28(71.8)	106(77.9)	11(12.0)
Negative	138(27.3)	3(20.0)	9(7.4)	45(44.1)	10(25.6)	27(19.9)	42(45.7)
No data	89(17.6)	0(0)	6(4.9)	40(39.2)	1(2.6)	3(2.2)	39(42.4)
Interferon-γ release assay							
Positive	291(57.5)	11(73.3)	106(86.9)	7(6.9)	36(92.3)	120(88.2)	13(14.1)
Negative	112(22.1)	3(20.0)	15(12.3)	46(45.1)	3(7.7)	14(10.3)	30(32.6)
No data	103(20.4)	1(6.7)	1(0.8)	49(48.0)	0(0)	2(1.5)	49(53.3)

*RTIs, respiratory tract infections; TB, tuberculosis*.

### Comparison of EasyNAT and Xpert

Among 239 children (137 PTB patients and 102 RTI patients) tested simultaneously with EasyNAT and Xpert, similar sensitivities were observed for both methods [22.6% (31/137) vs. 26.3% (36/137), respectively; *p* = 0.441]. Among 137 children with PTB, 15 (10.9%) were classified as bacteriologically confirmed TB and 122 (89.1%) were classified as probable TB. EasyNAT yielded identical sensitivity to Xpert for MTB detection in children with bacteriologically confirmed TB, with both assays demonstrating sensitivity of 60.0% (9/15). In children with probable TB, the sensitivity of Xpert was observed to be similar with that of EasyNAT, the difference was not significant [22.1% (27/122) vs. 18.0% (22/122), respectively; *p* = 0.424]. Both EasyNAT and Xpert methods showed high specificity in children with RTIs, demonstrating 98.0% (100/102) and 99.0% (101/102) specificity, respectively (*p* = 1.000; Table 2).

**Table 2:** Comparison of EasyNAT and Xpert in children with TB and RTIs.

Group	Sensitivity, % (*n* of *N*)		*p*	Specificity, % (*n* of *N*)		*p*
	EasyNAT	Xpert		EasyNAT	Xpert	
All enrolled children	22.6 (31of 137)	26.3 (36 of 137)	0.441	98.0 (100 of 102)	99.0 (101 of 102)	1.0
Bacteriologically confirmed	60.0(9 of 15)	60.0 (9 of 15)	1.0			
Probable TB	18.0 (22 of 122)	22.1 (27 of 122)	0.424			

*TB, pulmonary tuberculosis; RTIs, respiratory tract infections; EasyNAT, EasyNAT MTC assay; and Xpert, Xpert MTB/RIF*.

**Figure 2:**
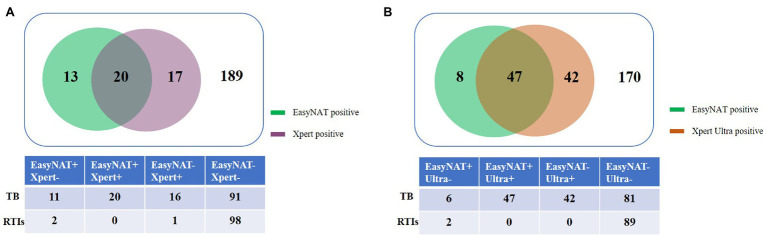
Venn diagram of the different diagnostic test results for childhood tuberculosis using gastric aspirate samples. **(A)** EasyNAT MTC and Xpert MTB/RIF. **(B)** EasyNAT MTC and Xpert MTB/RIF Ultra.

**Figure 3:**
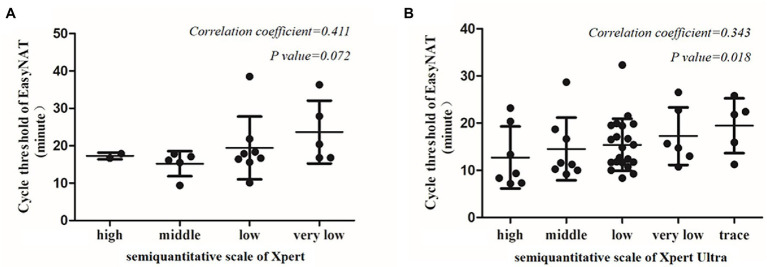
Correlation between the cycle threshold of EasyNAT MTC assay and the semiquantitative scale of Xpert MTB/RIF or Xpert MTB/RIF Ultra. **(A)** Correlation between EasyNAT MTC and Xpert MTB/RIF. **(B)** Correlation between EasyNAT MTC and Xpert MTB/RIF Ultra.

Agreement between EasyNAT and Xpert was moderate (κ = 0.498) among 239 children (Figure 2A). Concordant results were obtained for 209 children (20 cases positive by both tests [EasyNAT+ Xpert+]; 189 negative by both tests [EasyNAT− Xpert−]). Discordant results were found in 27 children with TB (16 EasyNAT− Xpert+ and 11 EasyNAT+ Xpert− results) and 3 children with RTIs (1 EasyNAT− Xpert+ and 2 EasyNAT+ Xpert− results). Among 27 children with TB, the semiquantitative scale of Xpert were low in 10 cases and very low in six cases, the cycle thresholds of EasyNAT were similar in children with EasyNAT+ Xpert− results than those with EasyNAT+ Xpert+ results (19.8 vs. 19.2, *p* = 0.844). Concordance of the semiquantitative scale for EasyNAT and Xpert was further analyzed for the 20 children with double positive results (Figure 3A). The results indicated a trend for patients with lower semiquantitative scores in Xpert being more likely to have higher cycle thresholds in EasyNAT, with a correlation coefficient of 0.411.

### Comparison of EasyNAT and Xpert Ultra

The detection outcomes of EasyNAT and Xpert Ultra assays among 267 children (including 175 children with PTB and 92 with RTIs) are presented in Table 3. Among 175 children with PTB, 39 (22.3%) were bacteriologically confirmed TB. Xpert Ultra showed higher sensitivity than EasyNAT, both in the total 175 PTB patients [50.9% (89/175) vs. 30.3% (53/175), respectively; *p* < 0.001] and in the 39 children with bacteriologically confirmed TB [82.1% (32/39) vs. 61.5% (24/39), respectively; *p* = 0.044], which indicated that Xpert Ultra was superior to EasyNAT despite its higher cost. If “trace” results in Xpert Ultra were not included, then the sensitivities of Xpert Ultra and EasyNAT were similar [34.9% (61/175) vs. 30.3% (53/175), respectively; *p* = 0.201]. Furthermore, similar specificities were observed in EasyNAT and Xpert Ultra assays, at 97.8% (90/92) and 100.0% (92/92), respectively (*p* = 0.155).

**Table 3:** Comparison of EasyNAT and Xpert ultra in children with TB and RTIs.

Group	Sensitivity, % (*n* of *N*)		*p*	Specificity, % (*n* of *N*)		*p*
	EasyNAT	Ultra		EasyNAT	Ultra	
All children	30.3 (53 of 175)	50.9 (89 of 175)	<0.001	97.8 (90 of 92)	100.0 (92 of 92)	0.155
Bacteriologically confirmed	61.5 (24 of 39)	82.1 (32 of 39)	0.044			
Probable TB	21.3 (29 of 136)	41.9 (57 of 136)	<0.001			

*Comparison of EasyNAT and Xpert Ultra in children with TB and RTIs. TB, pulmonary tuberculosis; RTIs, respiratory tract infections; EasyNAT, EasyNAT MTC assay; and Ultra, Xpert MTB/RIF Ultra*.

Agreement between EasyNAT and Xpert Ultra was further analyzed among 267 children, and concordant results were obtained for 217 children (47 EasyNAT+ Xpert Ultra+; 170 EasyNAT− Xpert Ultra−). Discordant results were found in 48 children with TB (42 with EasyNAT− Xpert Ultra+ and 6 with EasyNAT+ Xpert Ultra- results) and 2 children with RTIs (2 with EasyNAT+Xpert Ultra- results; Figure 2B). Among 48 children with TB, the semiquantitative scale of Xpert Ultra was low in 11 cases, very low in eight cases, and trace in 23 cases, indicating the bacterial load of these samples were low. The cycle thresholds of EasyNAT were higher in children with EasyNAT+ Xpert Ultra-results than those with EasyNAT+ Xpert Ultra+results (27.0 vs. 15.5, *p* = 0.083). Additionally, we found that among 28 Xpert Ultra trace cases, five were EasyNAT+.

Concordance of the semiquantitative scales for EasyNAT and Xpert Ultra was analyzed in 47 PTB children with both double positive results (Figure 3B). The result showed that patients with lower semiquantitative scores in Xpert Ultra tended to have higher cycle thresholds in EasyNAT, with a Spearman’s rank correlation coefficient of 0.343 (*p* = 0.018).

### Invalid Results From the Assays

Among all the enrolled children, none had an invalid Ultra test result. Three “invalid” results were initially obtained from the EasyNAT test; after repeated testing two were “positive” and one had “negative” results. Two “invalid” results were initially obtained from the Xpert test and the repeated resulted showed “positive” results.

## Discussion

Since 2010, the WHO has recommended that national health authorities employ the Xpert MTB/RIF assay in the management of TB, multi-drug resistant TB, and HIV-associated TB (World-Health-Organization, 2011b). The global TB report published in 2018 introduced the EasyNAT assay, which was developed in China as a possible alternative to the GeneXpert platform in primary health care facilities (World-Health-Organization, 2018). EasyNAT has a similar limit of detection (LOD) to Xpert [100 colony forming units (CFU)/ml and 114 CFU/ml, respectively]. However, evidence regarding the performance of EasyNAT is limited. No data indicating its diagnostic value in children have been published. Therefore, the global report highlighted that well-designed validation studies are needed to enable the WHO to review and assess the performance of this assay.

The first aim of the present study was to compare diagnostic accuracy between EasyNAT and Xpert or Xpert Ultra. Similar sensitivities and specificities were observed between the two assays. Furthermore, the semiquantitative scale of Xpert was also concordant with the cycle threshold generated by EasyNAT, indicating an equal detection efficiency in childhood TB using GA specimens. The sensitivities of EasyNAT and Xpert in bacteriologically confirmed TB were both 61.5%, which is similar to previously reported data from a meta-analysis (66.0%; Chakravorty et al., 2017). Considering the low cost of the EasyNAT assay, less than half that of Xpert, it may be a promising diagnostic assay for childhood TB.

The use of Xpert Ultra in GA and stool samples has been suggested for TB diagnosis in children (World-Health-Organization, 2021c). Because of the improved detection efficiency with a LOD of 16 CFU/ml, Xpert Ultra presented a higher sensitivity than EasyNAT (50.9% vs. 30.3%, respectively, *p* < 0.001) and Xpert (50.9% vs. 26.3%, respectively *p* < 0.001). The relative higher sensitivity of Xpert Ultra compared with Xpert in children has been reported in our previous studies, at 80 and 67%, respectively, using bronchoalveolar lavage fluid (Sun et al., 2019), and 66.7 and 42.9%, respectively, using sputum (Peng et al., 2021). To improve sensitivity for the detection of MTB, an additional two multicopy amplification targets (IS*6110* and IS*1081*) are used in Xpert Ultra. Because of the paucibacillary specimens obtained from children, 31.4% (28/89) of Xpert Ultra positive results were classified as “trace” in the semiquantitative scale in the present study, suggesting the value of Xpert Ultra in samples with low bacillary burden for MTB detection (Chakravorty et al., 2017). It suggested that Xpert Ultra performed better than EasyNAT in children although the current cost of the former hindered its implementation. In addition, we found among 28 cases with an Xpert Ultra trace readout, five were EasyNAT+. This suggested a promising use for EasyNAT in children with low bacterial burden samples.

We also found some discordant results between EasyNAT and the Xpert or Xpert Ultra. In children with RTIs, four with EasyNAT-positive and one with Xpert-positive results presented a very low load of MTB. All five children have no symptoms or signs or X-ray abnormalities suggestive of TB, no contact history of active TB, and positive responded to antibiotic treatment rather than anti-TB treatment. Among them, four children had negative IGRAs results, and one child was diagnosed as latent TB infection and *Mycoplasma pneumonia*. All the children with discordant results were not further tested with a third nucleic acid-based test because of the following reasons: First, some of these TB children (27/75) were confirmed by MTB culture, and the rest of the TB cases (48/75) were clinically diagnosed based their clinical manifestations, chest X-ray results, clinical presentation improvement after anti-TB treatment, and positive results of tuberculin skin test or interferon-γ release assay. Second, Xpert Ultra is considered to be the most sensitive nucleic acid detection test, with the lowest limit of detection of 15.6 CFU/ml. So the negative results of the tests in children with discordant results suggested a underdiagnosis in these cases.

The first generation EasyNAT TB Isothermal Amplification Diagnostic Kit (EasyNAT TB IAD) and the next-generation EasyNAT MTC assay used here have been evaluated in the diagnosis of TB in adults (Ou et al., 2014; Zhang et al., 2021). One study reported that the sensitivity of EasyNAT TB IAD in adults with culture-confirmed TB was 84.1% (Ou et al., 2014). The next-generation EasyNAT MTC assay performed in a clinical setting demonstrated an overall sensitivity of 72.19%, which further increased to 86.84% in cases with confirmed TB using sputum (Zhang et al., 2021). Compared with these findings, the sensitivity of EasyNAT was lower in children. Several reasons may have contributed to the differences in sensitivities between the adult subjects enrolled in previous studies and the present childhood cohort. Firstly, the bacillary burden of bacteria in adult specimens is higher than that in children, which may lead to a lower bacterial positivity rate in children compared with adults. According to nationwide data reported in Mainland China, the positivity rate of bacteriology in children was 21% (Yang et al., 2020), while the bacteriologically confirmed rate in adults was 37.5% (Pang et al., 2019). Thus, diagnosing TB in children is more difficult than in adults, highlighting the urgent need for high sensitivity nucleic acid amplification tests for diagnosis of childhood TB.

Secondly, the detection rates of molecular tests vary between different specimen types. Sputum, the main specimen type in adult PTB patients, is difficult to obtain in children. However, GA is an invasive but well-tolerated procedure that can be used in children. WHO Guidance for the management of tuberculosis in children suggested to use early-morning GA for pediatric TB diagnosis as children swallow respiratory secretions (World-Health-Organization, 2014). Therefore, GA is an alternative specimen type for children with unavailable sputum samples. Data from the German national notification system showed that GA samples were used in 59% of the children who were diagnosed with pulmonary TB, and GA was the only reported specimen for 34.7% of the bacteriological notified patients (Fiebig et al., 2014). One meta-analysis reported that expectorated sputum was the best sample type for diagnosing adult PTB, with a pooled sensitivity of 90%, while the pooled sensitivity of expectorated sputum in children was only 14%. Although GA specimens were suitable for childhood TB diagnosis, the sensitivity (80%) was slightly lower than that of sputum in adults (Lyu et al., 2020). The pretreatment protocol applied to GA specimens in this study was adapted from that of sputum samples; whether this method is suitable and can affect the detection rate of the EasyNAT MTC assay remains unclear. The processing methods for GA samples should be optimized in future studies.

In conclusion, the EasyNAT assay had a similar diagnostic capacity for MTB in GA samples compared with the Xpert assay in children but demonstrated a lower sensitivity than Xpert Ultra. EasyNAT may therefore be useful as a suitable alternative method of childhood TB diagnosis based on its cost-effectiveness, speed, and accuracy.

## Data Availability Statement

The raw data supporting the conclusions of this article will be made available by the authors, without undue reservation.

## Ethics Statement

The studies involving human participants were reviewed and approved by the Medical Ethics Committee of Beijing Children’s Hospital. Written informed consent to participate in this study was provided by the participants’ legal guardian/next of kin.

## Author Contributions

SQ, TJ, WJ, YW, and LS performed the tests. YZ, QL, YL, MF, YS, LD, XT, and XS enrolled the subjects and collected data and samples. SQ and LS analyzed the data and drafted the initial manuscript. CW, LS, and AS conceptualized and designed the study and reviewed and revised the manuscript. All authors contributed to the article and approved the submitted version.

## Funding

This work was partly supported by the Capital’s Funds for Health Improvement and Research (2020-1-2091), Key Project of the Department of Science and Technology Beijing, China (D181100000418003), and National Science and Technology Major Project of China (2018ZX10103001-003).

## Conflict of Interest

The authors declare that the research was conducted in the absence of any commercial or financial relationships that could be construed as a potential conflict of interest.

## Publisher’s Note

All claims expressed in this article are solely those of the authors and do not necessarily represent those of their affiliated organizations, or those of the publisher, the editors and the reviewers. Any product that may be evaluated in this article, or claim that may be made by its manufacturer, is not guaranteed or endorsed by the publisher.
