# Supplementary material for: A Novel Cross-Priming Amplification-Based Assay for Tuberculosis Diagnosis in Children Using Gastric Aspirate
Source: Front Microbiol. 2022 Mar 24;13:819654. doi: 10.3389/fmicb.2022.819654 (PMC8988679; doi:10.3389/fmicb.2022.819654)
Supplement: Supplementary file 1 [file Table_1.DOCX]

Supplementary table 1. Microbial pathogen identified in RTIs and probable TB group.

| Group | Microbial pathogens | Number |
| --- | --- | --- |
| RTIs | *Mycoplasma pneumoniae* | 92 |
|  | *Mycoplasma pneumoniae* and *Streptococcus pneumoniae* | 14 |
|  | *Mycoplasma pneumoniae* and Adenovirus | 12 |
|  | *Mycoplasma pneumoniae* and Respiratory syncytial virus | 8 |
|  | *Mycoplasma pneumoniae* and Boca virus | 6 |
|  | Respiratory syncytial virus | 15 |
|  | *Streptococcus pneumoniae* | 22 |
|  | *Streptococcus pneumoniae* and Haemophilus influenzae | 10 |
|  | *Staphylococcus aureus* and Haemophilus influenzae | 8 |
|  | Haemophilus influenzae and Methicillin-resistant *Staphylococcus aureus* | 7 |
| Probable TB | Mycoplasma pneumoniae | 15 |
|  | Adenovirus | 1 |
|  | Epstein-Barr virus | 1 |

RTIs, respiratory tract infections; TB, tuberculosis.
